# Non-invasive Assessment of Intracranial Pressure in Severe Burned Patients: From Animal Models to Bedside

**DOI:** 10.37825/2239-9747.1054

**Published:** 2024-07-25

**Authors:** Maria Notaro, Laura M.B. Belotti, Marco D. Serafino, Adele Longobardi, Romolo Villani, Raffaele Aspide

**Affiliations:** a AORN Antonio Cardarelli di Napoli, Burn Intensive Care Unit, 80131 Napoli, Italy; b IRCCS Istituto delle Scienze Neurologiche di Bologna, Epidemiology and Statistic Unit, 40139 Bologna, Italy; c AORN Antonio Cardarelli di Napoli, Department of General Emergency and Radiology, 80131 Napoli, Italy; d IRCCS Istituto delle Scienze Neurologiche di Bologna, Anesthesia and Neurointensive Care Unit, 40139 Bologna, Italy

**Keywords:** Intracranial pressure, Optic nerve sheath diameter, Transcranial Doppler, Burned patients, Non-invasive intracranial pressure, Critical patients

## Abstract

**Aims:**

Some is known from studies on burn animal models. Burn patients can develop intracranial hypertension. The aim of study is to evaluate feasibility of non-invasive methods for the diagnosis of intracranial hypertension.

**Methods:**

Burns patients were enrolled and studied through ultrasound measurement of optic nerve sheath diameter and transcranial Doppler.

**Results:**

In the 20 patients studied, no pathological values were identified without correlations with the extension of the burn.

**Conclusions:**

Starting from animal models, it is legitimate to suspect an underestimation of neuro-complications. The study demonstrates that these methods are applicable to this population, representing an effective method reducing the incidence of neuro-complications.

## 1. Introduction

Shock in the burn patient is due to the combination of hypovolemic shock and cellular shock, characterized by specific microvascular and hemodynamic alterations [1]. After injury, there is a significant loss of circulating plasma volume due to increased capillary permeability, which is derived from vascular injury and the release of inflammatory mediators [2]. As a result, edema emerges in both burned and unburned tissues, followed by the depletion of intravascular volume, reduced cardiac output and increments in systemic vascular resistance [2–6].

In humans as in the sheep models studied, cerebral circulation is an integral part of systemic circulation. However, the hemodynamic changes in cerebral circulation are significantly different from those in systemic circulation [2,7,8]. Such a difference is largely due to the autoregulation of cerebral blood flow, which is able to remain relatively constant despite changes in systemic perfusion pressure but only within certain limits [8]. In sheep model studies (the only ones available), burns may have a significant impact on the level of intracranial pressure (ICP) during resuscitation phase, while the cerebral perfusion pressure after an initial reduction undergoes an inappropriate increase, suggesting an alteration of autoregulation in the hours following the burn (after 6 h) [2,7]. The elevated ICP was more likely to be the result of encephaledema rather than arterial carbon dioxide tension, as it remained constant post-burn [7]. Although ICP can guide patient management in neurocritical care, it is not commonly monitored in many clinical conditions outside this setting. Provided that data of ICP can be crucial for the successful management of patients in many subcritical conditions [9], non-invasive estimation of ICP (nICP) method would be useful to determine whether a patient’s ICP is elevated or is changing [10–12]. Apart from many clinical applications, Transcranial color-coded duplex (TCCD) waveform analysis has been investigated as a technique for nICP estimation, and this could represent one of its most useful applications outside the neurocritical care setting. It is conceivable if one considers that increased ICP could affect the waveform of blood flow velocity (FV) in major cerebral vessels which have compliant walls. In addition, another nICP method can also be used: the evaluation of the optic nerve sheath diameter (ONSD) using ultrasound (US) which can detect the dilation of the optic nerve sheath, 3 mm behind the optic disk [13–15]. A prospective study suggested TCCD has a good negative predictive value, excluding elevated ICP [16], as a retrospective study have considered [13].

The primary aim of the present study was to evaluate the feasibility of real-time nICP measurement [9] in a cohort of burn patients when they accessed intensive burn care using TCCD and US ONSD and their correlation with burn size and mortality rate.

## 2. Methods

### 2.1. Study design

In this observational study, Authors analyzed prospectively collected data in severe burn patients that require admission to the burn intensive care unit (ICU) of A. Cardarelli Hospital, Naples, Italy, from October 2021 to December 2022. The study was conducted in accordance with the Declaration of Helsinki and the general principles of Good Clinical Practice. The Institutional Review Board of the AORN Cardarelli-Santobono approved the study to be conducted (CE 66/2019) Authors followed the Strengthening the Reporting of Observational Studies in Epidemiology (STROBE) guidelines for cohort studies (http://www.strobe-statement.org (accessed on February 20, 2023)).

### 2.2. Population

The following criteria were required for adult patient enrollment: age ≥18 years, admission to ICU, burn size ≥20% Total Body Surface Area (TBSA), deep dermal burns and full thickness burns and enrollment to the study within 8 h of injury. Patients with a diagnosis of idiopathic intracranial hypertension or pseudotumor, with diseases resulting in increased intracranial pressure, severe burn at the level of the temporal and optic nerve ultrasound windows, with prior severe head trauma, represented limiting factors to intracranial hemodynamic assessment and exclusion criteria for TCCD and ONSD examinations. Furthermore, those patients, guardian/family member/fiduciary who did not agree to sign the consent to participate in the study were excluded.

### 2.3. Data collection and definitions

In the present study the Authors collected demographic data, medical history, the acute physiology and chronic health evaluation and burn extension on admission. A severe burn injury is defined using standard criteria, in general for adults, it is severe any burn roughly >20% of the TBSA, excluding superficial burns (epidermal; first-degree burns) [17] results in acute systemic responses collectively known as burn shock [18].

The size of the burn is estimated using the “rule of Nines” method [19]. It’s a system used to predict the chance of mortality due to burn [20]. The proportion of patients who died (case fatality) is plotted as a function of the sum of age and the total percentage of body surface area (TBSA) burned [21]. The mortality rate increases with the size of the burn. The population analyzed was divided by mortality risk into three categories: moderate, intermediate and high (Table 1).

During the examination, patients were in the supine position with a head elevation of no more than 30°. Using TCCD with a Mindray ultrasound 2-MHz probe, blood FV in the middle cerebral artery (MCA) was monitored, like low diastolic cerebral blood flow velocity (FVd), mean flow velocity (FVm), and Pulsatility Index (PI) values can be observed with TCCD [9]. The diameter of the optic nerve sheath 3 mm behind the optic disk surface, was measured using an Mindray ultrasound 7.5-MHz probe [13]. The probe was applied with coupling gel to the closed eyelid.

Two measurements, sagittal and transverse, were taken for each optic nerve with standardized equipment settings using the digital cursor and measurement software of the ultrasound machine [13,22]. Data were collected exactly at time 0 (within 8 h of the burn), time 1 (48 h after) and time 2 (96 h after the burn).

**Fig. 1:**
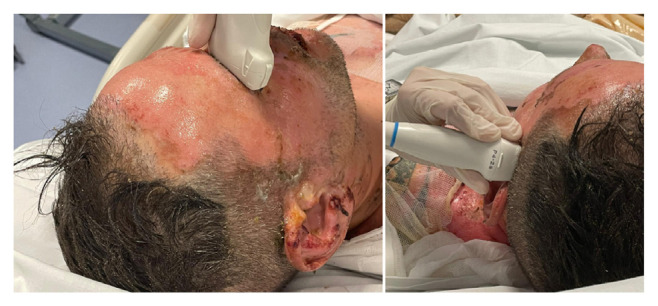
Two ONSD (left) and TCCD (right) measurement images on the head of a severely burned patient (80% extension) with “accessible ultrasound windows”, as they are not affected by the burn.

Are considered to be pathological FVd <20 cm/s, FVm <30 cm/s, PI >1.4; ONSD >5 mm is considered ICP >20 mmHg. The combination of at least two pathological values out of the three has been considered highly suggestive of intracranial hypertension (Fig. 1).

### 2.4. Study outcomes

The primary outcome of the study was to assess the occurrence of high-ICP episodes in burned patients using non-invasive evaluation (TCCD/ ONSD). The secondary outcomes were the correlations of TCCD and ONSD values with burn size and mortality rate.

### 2.5. Statistical analysis

The collected data will be analyzed descriptively with measures of centrality and variance (mean, median, standard deviation (SD) and interquartile range (IQR)), based on the distribution (normal or non-normal) of the data themselves. The ONSD values were used as averages between transverse and sagittal measurements and subsequent averages between left eye and right eye: an average ONSD value was thus obtained for each patient at each evaluation step. ONSD and TCCD parameters in the different burn groups were compared by the Mann–Whitney *U* test and Spearman’s rho for the non-parameter estimation of the correlation. Statistical analysis was performed using Stata statistical software version 16 (Stata Corp LLC, College Station, TX, USA).

## 3. Results

A total of 20 patients with burn size ≥20% TBSA, deep dermal and full thickness burns were admitted over the study period. 7 patients were female and the mean age of the patients was 56.8 years with a SD of ±23.78. Nine patients had TBSA between 20 and 30%, 7 had TBSA between 31 and 50%, 4 had TBSA between 51 and 90%. All patients receiving fluid therapy, of which 2 with extended burn between 30 and 50% and 4 between 50 and 90% were under mechanical ventilation and cardioactive medications (i.e. vasopressors and/or inotropic agents) at the time of TCCD and measurement of ONSD assessment.

### 3.1. TCCD FVd

The mean FVd values and SD at time 0, time 1 (48 h) and time 2 (96 h), distinguished by the three burn groups are described in detail in Table 2. Only two recorded values were lower than the critical threshold <20 cm/s in the time 0 and 2.

### 3.2. TCCD FVm

The mean FVm values and SD at time 0, time 1 (48 h) and time 2 (96 h), distinguished by the three burn groups are described in detail in Table 3. No recorded value was lower than the critical threshold <30 cm/s.

### 3.3. TCCD PI

The mean PI values and SD at time 0, time 1 (48 h) and time 2 (96 h), distinguished by the three burn groups are described in detail in Table 4. No recorded value was higher than the critical threshold >1.4.

### 3.4. ONSD

The mean ONSD values and SD at time 0, time 1 (48 h) and time 2 (96 h), distinguished by the three burn groups are described in detail in Table 5. No recorded value exceeded the threshold >5 mm.

### 3.5. Correlations

The FVd, FVm, PI and ONSD measurements in the 3 stages, although plausible and homogeneous, with particularly narrow SD and IQR, do not demonstrate statistical significance in non-parametric tests when related to burn extension/mortality rate. There is no association between these values in the various steps and the severity of the burn.

## 4. Discussion

In the present study, PI and FVd and FVm were not different in TBSA percentage and patients with partial and full thickness; these parameters have always been under our cut-off values. In the same way, the mean values ONSD at time 0, time 1 and time 2 have never been more than 5 mm. However, FVd in the single measurement at time 0 and 2 was found to be slightly under the cut-off values. Afterwards in time 1 the derivative data were again in the normal range. Obtained data on enrolled burn patients show the absence of significant alteration in ICP.

These data cannot be compared because Authors did not find any other in the scientific literature, but they highlight the feasibility of the ultrasound-based method.

Burn-related brain injury remains a critical issue that should not be neglected and moreover nICP estimation could be a precious support in neuro-worsing valuation algorithm. The burns studied in mice highlight the alteration of the blood-brain barrier, through mechanisms yet to be clarified, which certainly have a decisive role in neurological alterations [23]. Even minor burns revealed neurobehavioral alterations in the mice studied, the expression of permanent brain alterations [24].

In a report a retrospective, clinicopathologic study of 139 patients who died during treatment of a severe burn, fifty-three percent of the patients had central nervous system complications-infections, cerebral infarcts and hemorrhages, metabolic encephalopathies, central pontine myelinolysis, and cerebral trauma. Children and adults were equally affected [25].

From studies on animal models we know that alterations in ICP and autoregulation occur in the phase immediately following the burn. Although in a specialist environment such as burns patients, computed tomography of the brain is readily available, for patients who are often complex to transport and transfer and transport due to clinical conditions. Very often these patients require sedation in the ICU, making constant monitoring of neurological conditions much more difficult. In such a scenario, methods for nICP could improve clinical management of these conditions. With present study, The Authors attempted to transfer the knowledge coming from studies on animal models, proposing methods applied as a routine in a neurocritical care environment in an environment dedicated to burn patients.

In addition to the irrefutable negative predictive value of TCCD, there are also promising results on ONSD. Hansen at All compared the ultrasonographic ONSD with the opening ICP measured invasively with an intraventricular catheter in order to assess the accuracy of ONSD to predict raised ICP. The results of their study suggest that an ONSD cut off of 5.5 mm is a strong predictor of ICP of >20 mm Hg. In a similar study Kimberley et al. reported an ONSD cut off of 5.0 mm to predict an ICP >20 mm Hg [26].

The idea of this pilot study is to try to design a non-invasive neuromonitoring protocol in burn patients. For example, T0 describes the measurement within the first 8 h of the occurrence, that is the hours in which a systemic inflammatory response syndrome, sepsis, develops even in animal models [7]. During the systemic inflammatory response syndrome, sepsis, severe burn injury, however, physical barriers between the systemic circulation and the cerebral parenchyma can be seriously compromised [27]. Fluid resuscitation remains the cornerstone of early burn management. Adequate fluid administration is critical to the prevention of burn shock and other complications of thermal injury [28]. The rationale is to match fluid resuscitation with the gradual resolution of the widespread increased vascular permeability that begins around 8–12 h post burn [29] To demonstrate the applicability of this monitoring, an observational case-control study with a larger series of cases will be necessary.

## 5. Limitations

This is a single center study with a small number of burn patients. Authors estimated hemodynamic brain, early (first 96 h), thus not excluding the possibility of alterations later on during the ICU stay. Moreover, the study did not make a difference between stable systemic arterial pressure and patients with cardioactive drug regimens, respiratory parameters, ventilation settings and their modifications. Furthermore, the neurological conditions of the patients were not taken into account (patients alert, sedated, in coma): patients who are not in a spontaneous coma, hardly have even incipient intracranial hypertension.

## 6. Conclusion

Optimal management of the severely burned patient can only be achieved if the treating critical care personnel are intimately familiar with the systemic and cerebral pathophysiology following severe burn trauma, and adjust their treatment modalities accordingly. The cerebral circulation dynamics can be observed with such methods as nICP changes in time domain, and tracked in real-time in the clinical setting. This is one of the advantages of TCCD and ONSD and may become particularly useful as a primary assessment tool in Centers where ICP measurements are not routinely applied, or in patients in whom ICP monitoring is unavailable or may not be clearly indicated [9].

## Figures and Tables

**Table 1:** Correlation between burn extension and mortality.

%TBSA	Mortality Rate	Mortality risk
0.1–9.9	0.6	Moderate risk
10–19.9	2.7	
20–29.9	8.6	
30–39.9	16.8	Intermediate risk
40–49.9	28.2	
50–59.9	37.8	High risk
60–69.9	47.2	
70–79.9	56.9	
80–89.9	78.2	
>90	87.7	

**Table 2:** The mean FVd values and SD, median and IQR at time 0 (within 8 h), time 1 (48 h) and time 2 (96 h).

		Mean value	SD ±	Median	IQR	*p*-value
FVd cm/sec time 0	Burn group 1	36.05	6.85	36.4	30.3–38.3	0.621
	Burn group 2	31.55	10.06	33.2	22.3–40.2	
	Burn group 3	36.13	8.69	36.91	29.45–42.81	
FVd cm/sec time 1	Burn group 1	34.69	3.81	35.4	33.1–35.6	0.392
	Burn group 2	30.9	8.55	28	23.3–40.1	
	Burn group 3	36.92	11.76	36.7	27.25–46.6	
FVd cm/sec time 2	Burn group 1	31.65	5.20	33.3	27.6–34.3	0.550
	Burn group 2	27.01	11.73	30.5	23–35	
	Burn group 3	33.96	8.87	35.17	26.7–41.22	

FVd, Flow Velocity Diastolic; SD, standard deviation; IQR, InterQuartile.

**Table 3:** The mean FVm values and SD, median and IQR at time 0 (within 8 h), time 1 (48 h) and time 2 (96 h).

		Mean value	SD ±	Median	IQR	*p*-value
FVm cm/sec time 0	Burn group 1	68.81	6.18	68.5	65–72.1	0.893
	Burn group 2	56.29	7.96	68.2	62.4–74.3	
	Burn group 3	67.27	5.93	66.4	62.8–71.75	
FVm cm/sec time 1	Burn group 1	69.35	6.03	67.7	65.7–75.6	0.980
	Burn group 2	70.48	7.94	73.1	61.2–78.5	
	Burn group 3	70.05	7.63	69.35	63.95–76.15	
FVm cm/sec time 2	Burn group 1	70.91	6.36	72.2	66.5–75.2	0.704
	Burn group 2	65.4	15.15	73.2	60.3–74.3	
	Burn group 3	68.45	5.30	67.65	64.3–72.6	

PI, Pulsatility Index; SD, standard deviation; IQR, InterQuartile.

**Table 4:** The mean PI values and SD, median and IQR at time 0 (within 8 h), time 1 (48 h) and time 2 (96 h).

		Mean value	SD ±	Median	IQR	*p*-value
PI time 0	Burn group 1	0.96	0.13	0.99	0.95–1.05	0.282
	Burn group 2	0.92	0.2	0.85	0.77–1.2	
	Burn group 3	0.81	0.08	0.82	0.75–0.87	
PI time 1	Burn group 1	0.92	0.16	0.92	0.86–1	0.992
	Burn group 2	0.94	0.19	0.9	0.8–1.14	
	Burn group 3	0.94	0.12	0.89	0.87–1.01	
PI time 2	Burn group 1	0.91	0.16	0.85	0.8–1	0.354
	Burn group 2	0.95	0.16	0.9	0.89–1.14	
	Burn group 3	1.04	0.1	1.1	0.99–1.1	

PI, Pulsatility Index; SD, standard deviation; IQR, InterQuartile.

**Table 5:** The mean ONSD values and SD, median and IQR at time 0 (within 8 h), time 1 (48 h) and time 2 (96 h).

		Mean value	SD ±	Median	IQR	*p*-value
PI time 0	Burn group 1	3.85	0.52	3.78	3.75–4.02	0.719
	Burn group 2	3.79	0.66	3.8	3.27–4.15	
	Burn group 3	4.02	0.35	4.01	3.75–4.3	
PI time 1	Burn group 1	3.7	0.58	3.77	3.4–4.07	0.496
	Burn group 2	3.74	0.45	3.77	3.3–4.12	
	Burn group 3	4.04	0.36	4.1	3.76–4.32	
PI time 2	Burn group 1	3.86	0.47	3.9	3.82–3.95	0.555
	Burn group 2	3.68	0.52	3.52	3.2–4.02	
	Burn group 3	3.77	0.34	3.74	3.56–3.98	

ONSD, Optic Nerve Sheath Diameter; SD, standard deviation; IQR, InterQuartile.

## Data Availability

The data supporting this study’s findings are available from the corresponding author, RA, upon reasonable request.

## Abbreviation

ICP: intracranial pressure
nICP: non-invasive ICP
TCCD: Transcranial color-coded duplex
FV: flow velocity
ONSD: optic nerve sheath diameter
US: ultrasound
ICU: intensive care unit
STROBE: Strengthening the Reporting of Observational Studies in Epidemiology
TBSA: Total Body Surface Area
BSA: Body Surface Area
MCA: middle cerebral artery
FVd: diastolic flow velocity
FVm: mean flow velocity
PI: Pulsatily Index
SD: standard deviation
IQR: interquartile range
